# Supplementary material for: RIAM: A Universal Accessible Protocol for the Isolation of High Purity DNA from Various Soils and Other Humic Substances
Source: Methods Protoc. 2022 Dec 16;5(6):99. doi: 10.3390/mps5060099 (PMC9783177; doi:10.3390/mps5060099)
Supplement: Supplementary file 1 [file mps-05-00099-s001.zip › mps-2081821-SI.pdf]

Supplementary Material

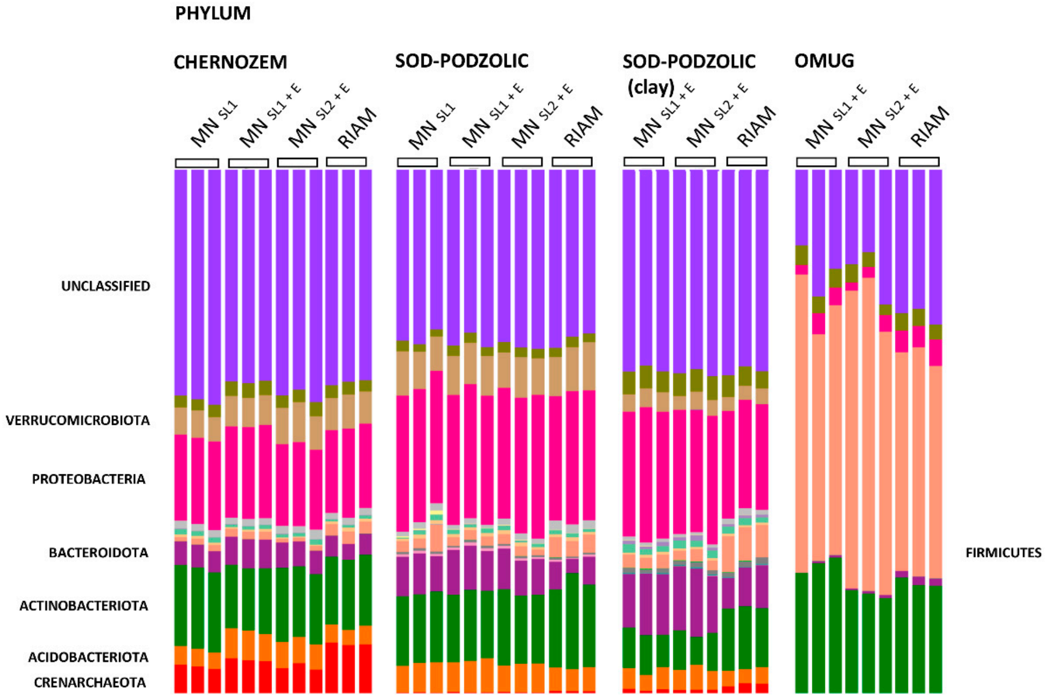

Figure S1. Taxonomy bargraphs, phylum level. Only major taxa named.

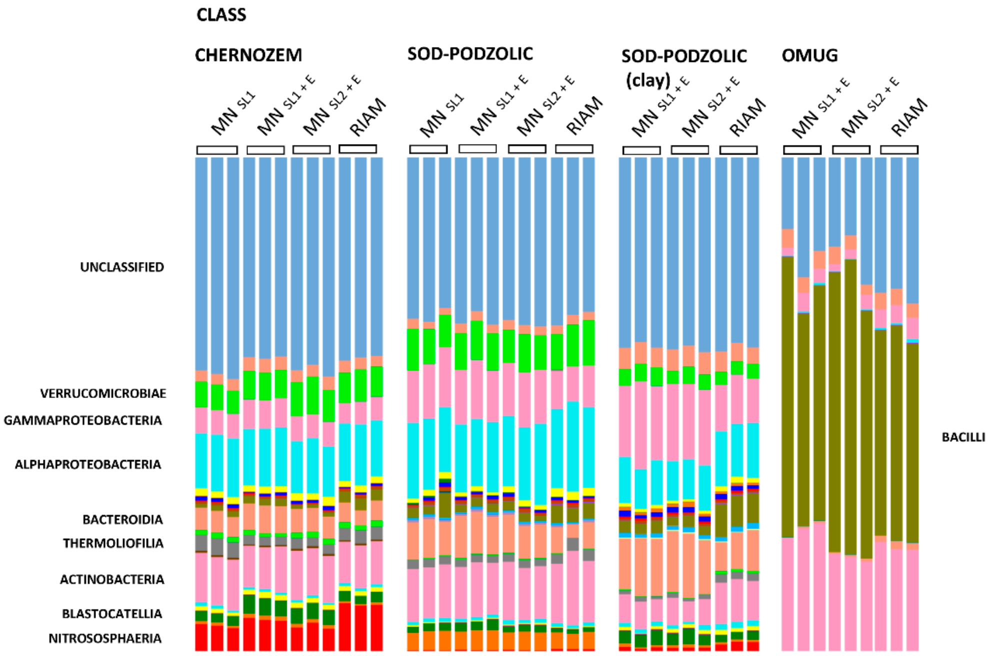

Figure S2. Taxonomy bargraphs, class level. Only major taxa named.

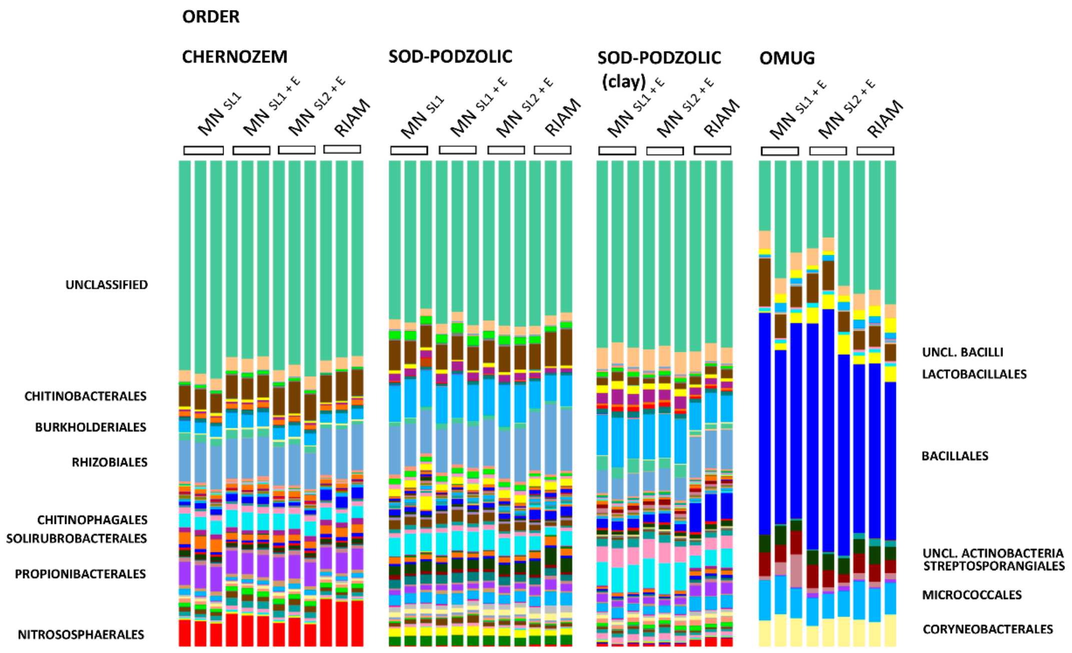

Figure S3. Taxonomy bargraphs, order level. Only major taxa named.

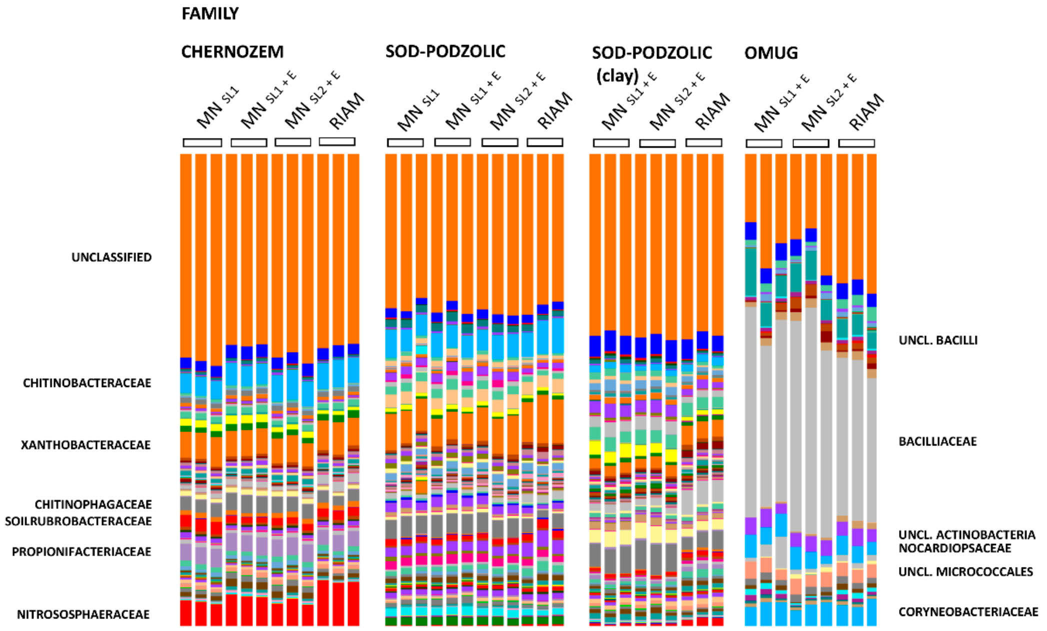

Figure S4. Taxonomy bargraphs, family level. Only major taxa named.

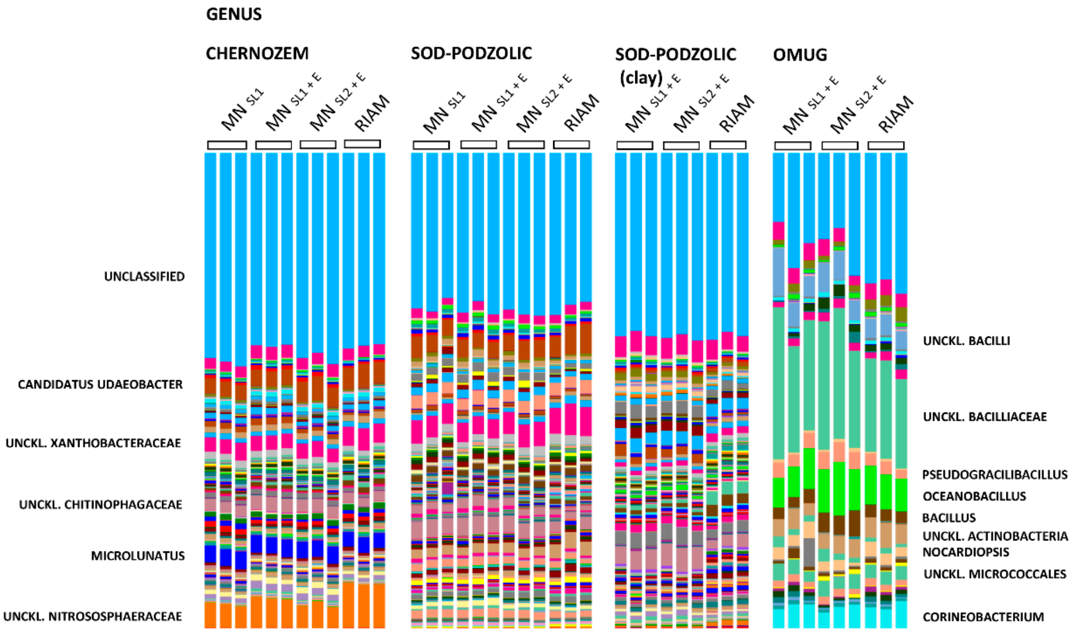

Figure S5. Taxonomy bargraphs, genus level. Only major taxa named.

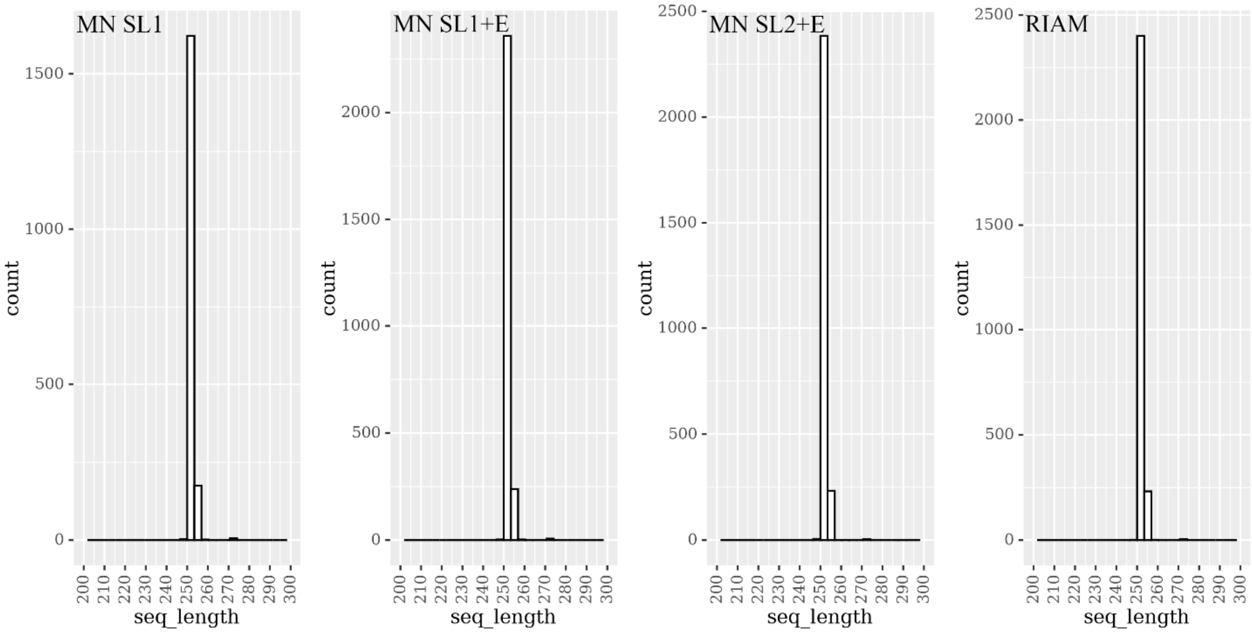

Figure S6. Sequence length distribution in 16S rRNA gene libraries.

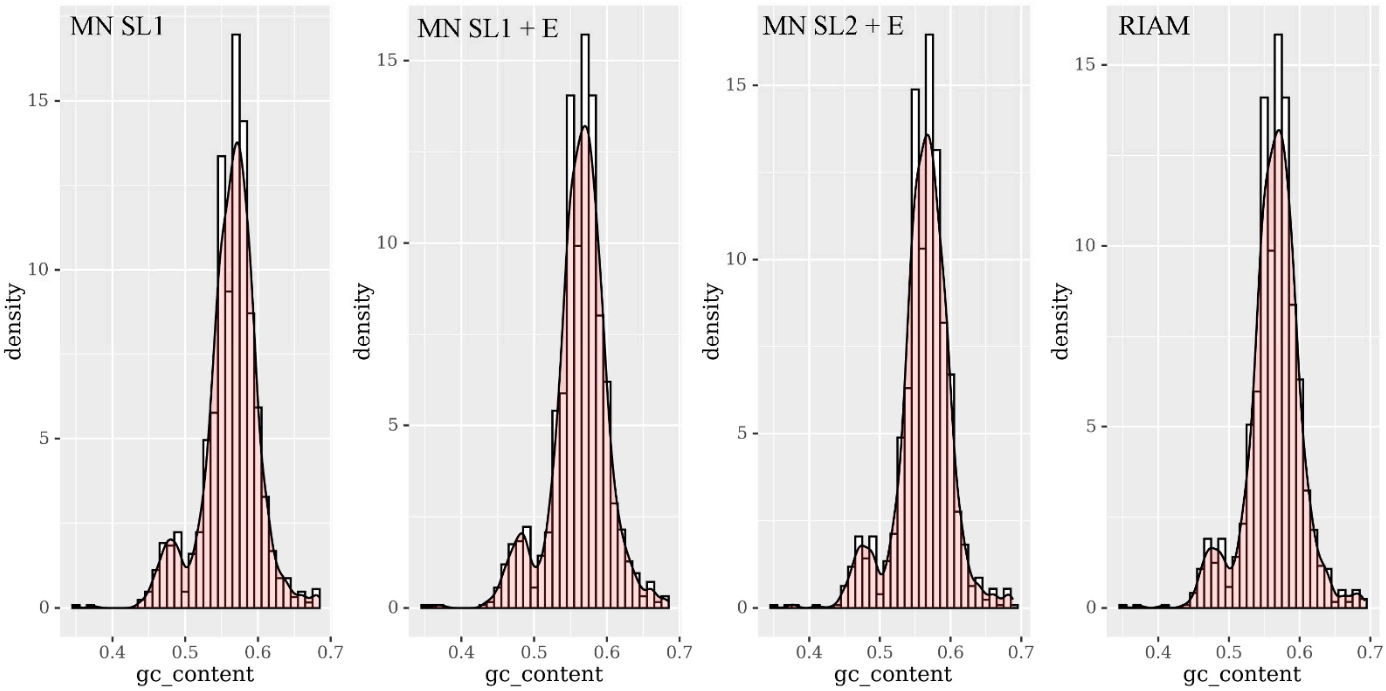

Figure S7. GC content distribution in 16S rRNA gene libraries.
